# Puerperal Convalescence and the Diseases of the Puerperal Period

**Published:** 1886-09

**Authors:** 


### Book Reviews.

Puerperal Convalescence and the Diseases of the Puerperal Period. *By* Joseph Kucher, M.D. *12mo, pp. 311. New York:* J. H. Vail & Co. *1886.*

Dr. Kucher has attempted in this book "to concisely reflect the views on the management of childbed, and upon the origin and treatment of the puerperal diseases, as accepted and in practice at the Vienna lying-in hospital." The purpose of the work is obviously excellent. The doctrines of the Vienna school have for a considerable period of time exercised an important influence upon the theory and practice of obstetrics in the United States, gradually replacing the English and French teachings of a former generation.

No adequate exposition of the puerperal diseases from the Vienna point of view has before appeared in English, and the lack of such a treatise has been keenly felt by every scientific practitioner of midwifery who is not familiar with the German language. The expectations of the general reader upon taking up Dr. Kucher's little book are high, and he is disposed to resent any imperfections in the performance of such an important undertaking. Then, too, Dr. Kucher has a large class of critics, composed of medical men, who have studied in Vienna and who ought to know the teachings of that school. Dr. Kucher's task is accordingly one of very unusual difficulty.

A careful reading of the book, however, must convince the most uncharitable critic that the design has been most admirably executed. More than this, Dr. Kucher adds value to the book by the free discussion of the relative values of the various methods of treatment. He writes as an authority upon the subject, and justly,—supported as he is by his experience in the Vienna lying-in hospital, a period of over four years, implying an observation of nearly 14,000 cases—and in private practice in New York.

In the brief space at our command, it is possible merely to call attention to a few of the topics considered.

In the chapter on "Mastitis," fissures and abrasions of the nipple are correctly mentioned as "the channels by which the infecting matter gains entrance to the breast and causes inflammation." While retention and impaired flow of milk, as ætiological factors, are relegated to their proper places among *a priori* conclusions and preconceived notions.

In the chapter on "Lacerations of the Perineum," the Vienna method of prevention is concisely sketched. Episiotomy—a little operation comparatively unknown in the United States—is recommended when laceration is otherwise unavoidable. In none of the more ambitious American or English text-books on midwifery has the Vienna method of perineal protection received such a clear description. It is a fact, an undeniable fact, that this distressing acccident seldom occurs in the Vienna lying-in hospital, while its relative frequency of occurrence in the United States is one of the *opprobria* of the art of midwifery. The accident is entirely avoidable in the very large majority of cases, and Dr. Kucher has clearly pointed out the way by which this object may be attained.

We notice, with regret, the absence of any suggestions with reference to the prevention of laceration of the cervix, also an accident of comparatively frequent occurrence in our country, and due, in many cases, to meddlesome midwifery, *i. e.*, the premature rupture of the bag of waters, the pernicious habit of digital dilatation, and the application of the forceps before complete dilatation of the cervix.

In the chapter on "Eclampsia," the theory of acute renal insufficiency is ably sustained. Pilocarpine and blood-letting are rightly relegated to the domain of obsolete measures.

About one-half of the book is devoted to the consideration of puerperal fever. The views of Semmelweiss are ably defended. All cases of puerperal fever arise "from the absorption of decomposing organic matter from lesions of the genital tract;" the septic element may be introduced from without (hetero-infection), or originated in the generative organs (auto-infection.) The note on "The Relation of the Atmosphere to Puerperal Fever" is of especial interest to the American practitioner, familiarized as he is with such vague, fanciful terms as "nosocomial malaria," "hospitalism," and the like. Dr. Kucher is very definitely of the opinion that atmospheric and telluric influences play a very insignificant role in the ætiology of puerperal fever. His views upon the subject do not differ essentially from the conclusions of Credé (*Gesunde und Kranke Wöchnerinnen, Leipzig, 1886.*) The doctrine of childbed fever, thus imperfectly indicated, is not attractive to the general obstetrician. As Dr. Kucher remarks: "Semmelweiss's theory is a blessing to the patient, but quite another thing to the physician. The old obstetricians were helpless in the prevention of puerperal fever, but they never had a sleepless night from remorse. There is nothing but the hard evidences of fact, *la brutalite des faits*, which forces us to accept this theory."

Dr. Kucher's little book, regarded as a whole, is, in our opinion, unquestionably the best treatise on the subject in the English language. It is one of the few books that are "to be chewed and digested;" that is, "to be read wholly, and with diligence and attention." W. W. J.

